# The Inflammation in the Cytopathology of Patients With Mucopolysaccharidoses- Immunomodulatory Drugs as an Approach to Therapy

**DOI:** 10.3389/fphar.2022.863667

**Published:** 2022-05-13

**Authors:** Anna-Maria Wiesinger, Brian Bigger, Roberto Giugliani, Maurizio Scarpa, Tobias Moser, Christina Lampe, Christoph Kampmann, Florian B. Lagler

**Affiliations:** ^1^ Institute of Congenital Metabolic Diseases, Paracelsus Medical University, Salzburg, Austria; ^2^ European Reference Network for Hereditary Metabolic Diseases, MetabERN, Udine, Italy; ^3^ Stem Cell and Neurotherapies, Division of Cell Matrix Biology and Regenerative Medicine, Faculty of Biology, Medicine and Health, University of Manchester, Manchester, United Kingdom; ^4^ Department of Genetics, Medical Genetics Service and Biodiscovery Laboratory, HCPA, UFRGS, Porto Alegre, Brazil; ^5^ Regional Coordinating Center for Rare Diseases, University Hospital Udine, Udine, Italy; ^6^ Department of Neurology, Christian Doppler University Hospital, Paracelsus Medical University, Salzburg, Austria; ^7^ Department of Child and Adolescent Medicine, Center of Rare Diseases, University Hospitals Giessen/Marburg, Giessen, Germany; ^8^ Department of Pediatric Cardiology, University Hospital Mainz, Mainz, Germany

**Keywords:** mucopolysaccharidoses, MPS, immunomodulation, inflammation, cytopathology, drug discovery

## Abstract

Mucopolysaccharidoses (MPS) are a group of lysosomal storage diseases (LSDs), characterized by the accumulation of glycosaminoglycans (GAGs). GAG storage-induced inflammatory processes are a driver of cytopathology in MPS and pharmacological immunomodulation can bring improvements in brain, cartilage and bone pathology in rodent models. This manuscript reviews current knowledge with regard to inflammation in MPS patients and provides hypotheses for the therapeutic use of immunomodulators in MPS. Thus, we aim to set the foundation for a rational repurposing of the discussed molecules to minimize the clinical unmet needs still remaining despite enzyme replacement therapy (ERT) and hematopoietic stem cell transplantation (HSCT).

## 1 Introduction

Mucopolysaccharidoses (MPSs) are a heterogeneous group of congenital lysosomal storage disorders (LSDs) caused by the deficiency in one of the enzymes involved in the degradation of glycosaminoglycans (GAGs). These macromolecules provide structural support to the extracellular matrix (ECM) and are involved in the cellular regulation and communication processes (Sepuru and Rajarathnam, 2019). MPSs are classified into 7 main types and several subtypes, related to 11 specific enzyme deficiencies (Neufeld and Muenzer, 2001). Although the clinical features exhibited by MPS patients differ depending on species of GAGs accumulated, reduced life expectancy is present in all types. Clinical symptoms, age of presentation onset, diagnosis, treatment and complications vary from one MPS to another and even within the spectrum of the same MPS type. For this reason, transdisciplinary work to diagnose, treat and support is required.

At this point, only enzyme replacement therapy (ERT) and hematopoietic stem cell transplantation (HSCT) are available for the treatment of patients in a limited number of MPS types. Although ERT and HSCT are causal therapies, neither are curative and show several limitations (Lagler, 2018). ERT effects, particularly on bone and CNS pathology is very limited, as bioavailability in these target tissues is low (Chen et al., 2019). HSCT is only confirmed to be effective in the CNS in early detected cases of MPS I. For patients with other MPS types, evidence of efficacy is still limited or absent (Beck, 2007). There is no approved therapeutic option for patients with MPS IIIA-D, MPS IVB or MPS IX. Thus, additional therapeutic strategies are urgently needed.

Inflammation, especially neuroinflammation, has been reported in several MPSs (Parker and Bigger, 2018; Viana et al., 2020).

Immunomodulators are promising treatment options that may be used to cover clinical needs which are unmet with ERT. These molecules are not directed towards correction of the enzymatic defects and the causative gene mutation, but rather targeted to pathways that are secondarily altered in the MPS. This dysregulation may be pharmacologically addressed by anti-inflammatory therapies to be used along with ERT or as monotherapy in types where approved ERTs are missing.

Acetylsalicylate and prednisolone have been tested in the treatment of inflammation in MPS. Although in both animal studies a significant reduction in cytokines and oxidative stress have been observed, such high doses of anti-inflammatory drugs are hardly feasible in humans, given the adverse effects of long-term use of steroids or COX inhibitors (DiRosario et al., 2009; Arfi et al., 2011).

Therefore, a better understanding of the underlying cell mechanism might improve the current knowledge about MPS and set the path for emerging treatments.

*In vivo* and *in vitro* studies have shown, that the toll-like receptor-4 (TLR4) pathway with downstream activation of the myeloid differentiation primary response 88 (MyD88) adaptor protein triggers neuroinflammatory processes in the brain of MPS patients. This results in production and release of pro-inflammatory cytokines (e.g., TNF-α and IL-1β) and chemokines (e.g., CXCR4 and MIP-1α) (Ausseil et al., 2008; Goodall et al., 2014). This inflammatory mechanism is mainly triggered by accumulation of the GAG heparan sulfate (HS) e.g., in MPS I, II and III but secondary storage molecules such as GM gangliosides may also trigger these pathways (Parker et al., 2020). It is likely to have a prominent impact on inflammatory processes in neurons and astrocytes (Goodall et al., 2014; Parker and Bigger, 2018). Therefore, in these forms of MPS, HS storage and TLR4 appear to be key drivers of neuropathy.

Moreover, an accumulation of the other GAGs namely DS (dermatan sulfate), KS (keratan sulfate), C6S (chondroitin 6 sulfate), C4S (chondroitin 4 sulfate) and HA (hyaluronan) might contribute to lysosomal dysfunction and secondary events, such as abnormal vesicle and plasma membrane trafficking, impaired autophagy, mitochondrial dysfunction, oxidative stress, impaired Calcium (Ca^2+^) homeostasis with membrane permeabilization, lysosomal disruption and ultimately activation of the NLRP3 inflammasome (Parker and Bigger, 2018). The latter appears to have a dominating role in the induction of inflammation in MPS.

The scope of this article is to review current knowledge on inflammatory immune response in MPS and to deduct possible treatment strategies. We focus on market-approved drugs, which may be repurposed to be used in MPS patients.

### 1.1 Hurler/-Hurler Scheie/-Scheie Syndrome (MPS I) and Hunter Syndrome (MPS II)

#### 1.1.1 Metabolic Dysfunction

MPS I is caused by a deficiency of the enzyme α-L-iduronidase (IDUA) leading to the accumulation of DS and HS in lysosomes and the extracellular matrix (ECM) (Muenzer, 2011; Campos and Monaga, 2012). MPS I presents as a spectrum of phenotypes from attenuated to severe with many phenotypes in between. Three MPS I subtypes have been classified that differ in severity and onset of disease, usually classified as attenuate (Scheie Syndrome), intermediate (Hurler-Scheie Syndrome) and severe (Hurler Syndrome) (Zhou et al., 2020). The severe form, Hurler Syndrome, is related to absence or extremely low functional levels of IDUA activity, associated with genotypes such as deletions and nonsense mutations (Ahmed et al., 2014).

MPS II is caused by a deficiency of the enzyme iduronidate-2-sulfatase (IDS), ultimately leading to the accumulation of the same GAGs as in MPS I—DS and HS (Muenzer, 2011). Depending on the residual functional IDS activity, MPS II can present as a neuronopathic and a non-neuronopatic form. Two thirds of the affected MPS II patients present the severe neuronopathic form (Neufeld and Muenzer, 2001).

Due to the fact, that an accumulation of the same GAGs in MPS I and MPS II is present, the cytopathology and appearance of clinical manifestations is comparable within these two types of MPS. Therefore, biochemical mechanisms as well as the inflammatory immune response refer in MPS I and MPS II to the prominent role of HS and DS and its proteoglycans (Parker and Bigger, 2018).

On a microscopic scale, MPS is characterized by an existence of foamy (GAG-laden) macrophages, fibroblasts, bone, muscle cells and neural cells. Murine models show substantial accumulation of HS and DS in the liver, kidney, spleen, heart, and to a less extent in the CNS cells, already during the fetal period (Wang et al., 2010). The brain GAG storage may occur later and slower than in other tissues. This has been suggested by Holley et al. (2011), due to the measurement of HS levels in IDUA deficient mice tissues, whereby a minor storage in the brain compared with the liver was reported.

Deposits of extracellular GAG and foamy cells leading to an higher absorption of water in the tissue, thus the tissue become inflated. These also interfere with the structure of fibers like collagen and elastin (Hampe et al., 2020) with abnormal collagen IV deposition in basement membranes of adenoid and tonsillar tissues seen in patients resulting in ECM remodeling (Pal et al., 2018). Perturbations of the balance of DS against other ECM components is likely one of the underlying reasons for airway remodeling seen in these diseases.

DS-containing proteoglycans, decorin and biglycan, have collagen binding sequences and controlling functions in morphology, size, growth and content of collagen fiber (Reed and Iozzo, 2002). Decorin limits the diameter of collagen fibrils and a deficiency is associated with fragile skin and thin dermis, weak tendons, decreased airway resistance, slow wound healing process and delayed angiogenesis (Young et al., 2002; Maccarana et al., 2009). Biglycan has the ability to modulate bone-morphogenic protein 4 (BMP-4)-induced osteoblast differentiation and blocks BMP-4 activity, thus biglycan appears as an essential regulator in skeletal growth (Xu et al., 1998; Moreno et al., 2005). Moreover, biglycan affects the Wnt signaling pathway (Berendsen et al., 2011). A dysregulation of the Shh and Wnt/β-catenin signaling causing abnormal heart development and atrioventricular valve formation, demonstrated by a MPS II zebrafish model (Costa et al., 2017). DS-containing proteoglycans may be linked as well to cardiac manifestations and other vascular clinical features, due to a disruption of elastin fibers, resulting in elastin which is reduced in content and aberrant in structure (Hinek and Wilson, 2000).

HS proteoglycans are associated with the cell surface, syndecan and glypican, or the peri-cellular matrix, betaglycan and perlecan. ECM-associated HS proteoglycans interact with growth factors, growth factor receptors, collagen and other ECM proteins and are essential for the structural constituent of basal lamina. Syndecan and glypican interact with ECM components or cytoskeleton, including collagen and fibronectin *via* its extracellular GAG unit. Furthermore, they regulate the biological activity of ligands and act as a co-receptor to catalyze the interaction between ligand and receptor. Therefore, HS and its proteoglycans are significantly involved in the regulation of chemokine and cytokine gradients produced by cells that have been stimulated *via* pro-inflammatory cytokines (Bernfield et al., 1999; Pasquale and Pavone, 2019).

Severe signs of CNS disease in MPS I and MPS II patients, like cognitive decline, loss of speech, and behavior changes such as hyperactivity, directly correlate with HS levels in urine, blood and fibroblasts. Based on that, studies have shown higher HS levels for neuronopathic MPS I and MPS II patients (Wilkinson et al., 2012; Bigger et al., 2018). Thus, HS may be the key driver for neuroinflammation.

#### 1.1.2 Inflammatory Immune Responses

The accumulation of these substrates has been associated with cell-to-cell and cell-to-ECM adhesion leading to widespread inflammation and tissue damage (Hampe et al., 2020). The inflammatory reactions within the CNS and joints may be triggered primary and secondary storage of undegraded substrates. Several complex processes lead to lysosomal disruption and the final initiation of the NLRP3 inflammasome: 1) activation of the TLR4 pathway, 2) sequestering of immune cells in the ECM, 3) abnormal vesicle trafficking, 4) impaired autophagy, 5) mitochondrial dysfunction, 6) oxidative stress, 7) impaired Ca^2+^ homeostasis and membrane permeabilization.

**FIGURE 1:**
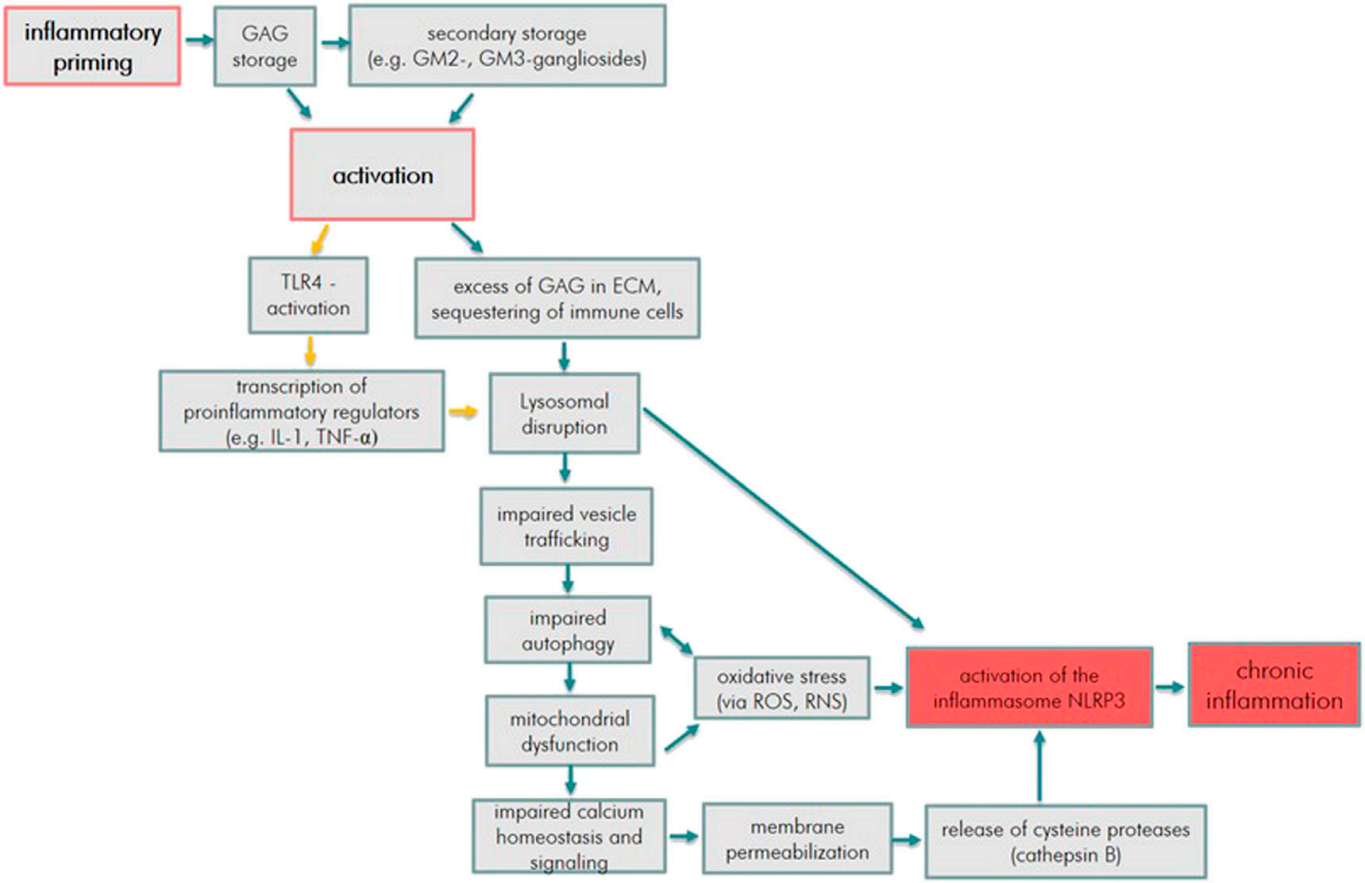
Inflammatory pathway cue to GAG storage in MPS (orange arrows indicate the process mainly due to HS accumulation, blue arrows indicate processes due to HS, DS, C4S, C6S, KS, and HA accumulation).

These mechanisms are illustrated in Figure 1 and primarily resemble the pathomechanism in MPS I and II. However, some aspects apply for all MPS types. In the sections about the other types, the main specificities described are the ones which differ from MPS I and II. The final activation of the inflammasome describes the instigation of long-term chronic inflammatory processes and cell death *via* pyroptosis.

In numerous LSDs, including MPSs, secondary storage of substances that is not described by the primary lysosomal defect has been consistently recognized and published (Parenti et al., 2021). The secondary storage of HS also occurs in the ECM and in the Golgi secretory pathway and acts as a positive regulator of HS-sulfation with the consequent accumulation of abnormal HS molecules at non-lysosomal sites. Viana et al. (2020) conducted a comprehensive analysis of brain cortex tissues from eight post-mortem autopsy sample of patients with MPS I, MPS II and MPS III and age-matched controls *via* high performance liquid chromatography (HPLC) together with histochemical staining of fixed tissues. A significant increase of HS and an accumulation of secondary substrates including GM2 and GM3 gangliosides has been described. The altered metabolism of gangliosides occurs very early in the course of the disease and may constitute a causal factor determining the progress of the CNS dysfunction. The accumulation of GM2 and GM3 gangliosides, as well as free cholesterol has been recognized to occur in patient’s neurons and animal models of MPS. In general, cholesterol and gangliosides are co-localized to specialized membrane micro domains, known as rafts, which are essential for cell signaling supposed. Their co-sequestration reportedly, could severely impact neuronal function and disease pathogenesis (Walkley, 2004; McGlynn et al., 2004). GM gangliosides are able to activate TLR4, and cholesterol is able to activate the inflammasome (Parker et al., 2020).

The mechanisms leading to secondary storage are not yet clarified. Basically, secondary storage might be caused by an inhibition by primary substrates of other lysosomal enzymes, changes of the lysosomal environment (e.g., pH changes) or an impairment of vesicle trafficking through the endosomal/lysosomal system and the autophagic pathway (Sobo et al., 2007; Fecarotta et al., 2020). Current studies suggest that secondary storage plays a major role in the pathophysiology of MPS and that this pathological mechanism is common for all MPS types, due to the fact that the post-mortem results by Viana et al. (2020) are similar to those in mouse models.

Especially the induction of TLR4 seems to play a major part in the pathogenic pathway of MPS. HS chains and proteoglycans are able to promote an inflammatory response *via* TLR4 activation, requiring CD44 and Myd88 (Simonaro et al., 2010; Parker and Bigger, 2018). Goodall et al. (2014) identified soluble HS fragments, which are released from the ECM, as TLR4 agonists, due to their LPS similar structure. Many aspects of HS interactions with TLR4 remain unclear. However, it is obvious that HS has a prominent role in facilitating innate immune responses, including a production of pro-inflammatory cytokines and chemokines (Parker and Bigger, 2018). The role of inflammatory and immune processes in the pathophysiology of CNS-, osteoarticular- and cardiovascular symptoms may largely been driven *via* TLR4 induction (Wilkinson et al., 2012; Opoka-Winiarska et al., 2013; Khalid et al., 2016; Hampe et al., 2020).

Based on these assumptions, Raymond et al. (2016) evaluated the cerebrospinal fluid (CSF) of 25 consecutive patients with MPS I Hurler. The cytokine analyses demonstrated a significant elevation of inflammatory markers including: IL-1β, TNF-α, MCP-1, SDF-1α, IL-1Ra, MIP-1β, IL-8, and VEGF in comparison to unaffected children.

Fujitsuka et al. (2019) measured for the first time eight biomarkers which were significantly elevated in untreated MPS II patients, compared to normal controls: EGF, IL-1β, IL-6, HS0S, HSNS, DS, mono-sulfated KS, and di-sulfated KS. Therefore blood samples have been collected from 46 MPS II patients. To understand the CNS pathology in the neuronopathic form of MSP II Bhalla et al. (2020) utilized a mouse model. In addition to the accumulation of CSF GAGs, neuronopathic MPS II patients showed elevated levels of lysosomal lipids, neurofilament light chain (NfL), and other biomarkers of neuronal damage and degeneration. Furthermore, they suggest that these biomarkers of downstream pathology are strongly correlated with HS. This is in agreement with the observation mentioned above, that plasma and CSF from MPS I patients showed significantly elevated inflammatory cytokines, including IL-1β, TNF-α, MCP-1, SDF-1α, IL-1Ra, MIP-1β, IL-8, and VEGF (Fujitsuka et al., 2019; Fecarotta et al., 2020).

It is assumed that TNF-α is the major driver and controller of peripheral inflammation in MPS. However, in the brain TNF-α is subordinated to other cytokine responses, such as IL-1. Nevertheless, there is no doubt that peripheral inflammation can influence central events. Therefore, targeted pharmacological strategies should address both central and peripheral inflammation (Parker and Bigger, 2018).

Several factors contribute to signaling dysregulation and sequestering of immune cells in the ECM. One of them is the synthesis of aberrant GAGs that disrupts physiologic GAG interactions with different receptors. The accumulation of GAGs and its incomplete degradation is not restricted to the lysosomes, but also occurs in the ECM. Furthermore, HS storage has also been detected in the Golgi secretory pathway, acting as a positive regulator of HS-sulfation and increasing the N-sulfotransferase activity of HS-modifying N-deacetylase/N-sulfotransferase enzymes (Campos and Monaga, 2012). Partially degraded HS with abnormal sulfation patterns has an impact on leukocyte and immune cell migration, further exacerbating inflammation (Hampe et al., 2020). These non-natural HS molecules, which contain increased sulfation and a non-reducing end, could impair cell response to different growth factors or cytokines, such as the fibroblast growth factors (FGFs) or bone morphogenetic proteins (BMPs) (Pan et al., 2005; Ballabio and Gieselmann, 2009; Holley et al., 2011). Significantly increased amounts and sulfation patterning of HS have been observed in brains of MPS I, MPS II and MPS III mice (Holley et al., 2011; Wilkinson et al., 2012; Bigger et al., 2018).

These GAGs were shown to modulate BMP-4 signaling activity in MPS I cells and to affect FGF2-HS interactions and FGF signaling in multipotent adult progenitor cells derived from MPS I patients (Pan et al., 2005). Dysregulated FGF-2 signaling was also found in MPS I chondrocytes, together with transformed GAG (Kingma et al., 2016).

ECM proteins, such as biglycan, fibromodulin, PRELP, type I collagen, lactotransferrin, and SERPINF1 were significantly reduced in the mouse model of MPS I, and further analysis identified several dysregulated mRNAs (e.g., Adamts12, Aspn, Chad, Col2a1, Col9a1, Hapln4, Lum, Matn1, Mmp3, Ogn, Omd, P4ha2, Prelp, and Rab32) (Heppner et al., 2015; Fecarotta et al., 2020). These modifications in the key structure may have a great impact in the pathogenesis of MPS I patients. FGF-2 acts as a proliferative agent and protector of several cell types, including neurons and their precursor cells (Alzheimer and Werner, 2003).

Sequestering hematopoietic stem cells to the ECM of bone marrow cells and limiting their migration has been demonstrated in MPS I and may occur due to an excessive 2-O-sulfatasion of HS and increased binding to the chemokine CXCL12 (Wilkinson et al., 2012). As all chemokines and cytokines have possible HS binding sites, this might has an impact on chemokine and cytokine signaling in the brain (Bigger et al., 2018).

In MPS II partially degraded HS has an increased level of sulfation on the carbohydrate backbone (Węgrzyn et al., 2010). Therefore, experts hypothesized that these modifications interfere differently with neuronal functioning, leading to differences in behavioral problems. However it is more likely that overly sulfated HS results in increased TLR4 activation and consequential activation of downstreaming signaling pathways to achieve this (Bigger et al., 2018).

Mitochondrial dysfunction caused by impaired autophagy has been recognized in several LSDs, such as sphingolipidoses (Gaucher disease, Niemann-Pick disease type C, Krabbe disease), gangliosidoses, multiple sulfatase deficiency and neuronal ceroid lipofuscinoses (Parenti et al., 2021) and proposed as one of the mechanisms underlying MPS neurodegeneration (Saffari et al., 2017; Annunziata et al., 2018). Findings were observed post-mortem in a MPS I patients brain, in particular in the cerebral limbic system, central gray matter and pons (Kobayashi et al., 2018). Another link between autophagy and MPS has been reported recently. Mutations in the *VPS33A* gene, encoding a protein which is involved in autophagy, resulted in an MPS-like disorder characterized by elevated levels of HS in plasma and urine, and in the main phenotypical MPS features (Kondo et al., 2016).

Impaired autophagy appears to be associated with dysregulation of the mechanistic target of rapamycin complex 1 (mTORC1) and AMP-activated kinase (AMPK) signaling (Lim et al., 2017; Pasquale et al., 2020; Stepien et al., 2020). It has been proven that autophagy controls IL-1β secretion by targeting pro- IL-1β for degradation and it has been extensively proven that there is a progressive block of autophagy in lysosomal storage disorders, including MPS (Azambuja et al., 2020). Furthermore, it has been observed that IL-1β and TNF-α correlate with each other. The correlation observed in this study by Jacques et al. (2016) suggests a possible involvement of NO in the induction and maintenance of inflammatory states in MPS II patient. Their results indicate that, at some extent, inflammatory processes, oxidative and nitrative imbalances are predominant in MPS patients—even during long-term ERT.

Oxidative stress has been identified as consequence of defects in mitophagy and mitochondrial dysfunction. These processes have been implicated in the pathogenesis of many neurodegenerative diseases, which share several features of neuroinflammation and cell death (Rego and Oliveira, 2003). An elevation of reactive oxygen and nitrogen species (ROS, RNS) by phagocytes and an accumulation of damaged mitochondria has been studied in MPS I animal models (Donida et al., 2015) and MPS I (Pereira et al., 2008) and MPS II (Filippon et al., 2011) blood samples. The patient samples demonstrated oxidative damage to proteins and lipids, increased catalase activity and reduced total antioxidant status—even under ERT. Interestingly, elevated levels of glutathione, responsible for the elimination of toxic peroxides and malondialdehyde, an indicator of lipid peroxidation, compared with control subjects have been presented (Pereira et al., 2008). This in turn, may contribute to inflammatory processes by a misidentification and autoimmune response to proteins damaged by oxidation—maybe even in the pathophysiology of bone and joint disease in MPS.

Unfolded protein response (UPR) and consecutive endoplasmatic reticulum (ER) stress may further contribute to cell disruption (Filippon et al., 2011; Pierzynowska et al., 2021).

Moreover, mitochondrial dysfunction can impair lysosomal functions, like acidification by the acidic pump V-ATPase, relying on the ATP generated by the mitochondria (Stepien et al., 2020). The mechanism inducing the rise in Ca^2+^ channel expression is still unclear, however it might be caused by an incomplete cellular glucose availability impairing mTORC1 activity of and resulting in increased Ca^2+^ channel gene transcription (Lim et al., 2015). Abnormal Ca^2+^ signaling may trigger the permeabilization of lysosomal membranes and allows an elevation of pH in the organelles impacting the activity and release of other lysosomal hydrolases into the cytoplasm, such as cathepsin B. (Boya and Kroemer, 2008; Pereira et al., 2010). This may trigger inflammatory signaling pathways, especially inflammasome activation and mitochondria dependent processes.

The relationship between autophagy and inflammation has recently been linked to inflammasome interactions in different medical situation and a subsequent release of the highly pro-inflammatory cytokine IL-1β (Abdelaziz et al., 2015). The activation of the NLRP3 inflammasome is mediated by the innate immune response to cellular stress signals such as lysosomal dysfunction, impaired ion homeostasis, free radicals, oxidative stress and other stimuli like ATP, leading to the maturation and release of IL-1β (Latz et al., 2013). An activation of ATP and most other NLRP3 activators leading to an efflux of K^+^. The intracellular Ca^2+^ increase, from intracellular stores or *via* membrane transporters, might be related to an activation of the NLRP3 inflammasome and the production and release of ROS might be another potential driver of inflammasome activation. Lastly, Bigger and Parker postulated that particulate matter taken up *via* phagocytosis can lead to lysosomal membrane permeabilization, the leakage of cathepsin B and other cysteine proteases and NLRP3 inflammasome activation (Parker and Bigger, 2018).

Gonzalez and colleagues have already demonstrated an overexpression of cathepsin B in MPS I mice (Gonzalez et al., 2018). In addition to that, Azambuja et al. (2020) observed in MPS II high levels of cathepsin B in the brain tissue and a leakage of the enzyme IDS, which is a known activator of the inflammasome. Furthermore, the author studied MPS II mice brains and demonstrated elevated activity of Caspase-1 and IL-1β, confirming that this pathway is indeed altered. However, no increase in NLRP3 levels was seen, it may be mediated either by other inflammasome proteins (such as NRLP1) or even *via* other pathways.

The interplay of these processes add up to the inflammation-induced cytopathology as illustrated in Figure 1.

### 1.2 Sanfilippo Syndrome (MPS III)

#### 1.2.1 Metabolic Dysfunction

MPS III is caused by a deficiency of one out of four different enzymes: N-sulfoglucosamine sulfohydrolase (MPS IIIA), α-N-acetylglucosaminidase (MPS IIIB), heparan-α-glucosaminide-N- acetyltransferase (MPS IIIC), N-acetylglucosamine-6-sulfatse (MPS IIID). The deficiency of any of these enzymes leads to the accumulation of HS in lysosomes and ECM (Muenzer, 2011). Subtype A is the most frequent subtype in European and North American countries. Therefore, more studies are linked to MPS IIIA.

In contrast to MPS I and II, in MPS III DS is only slightly accumulated. This dominance of the neurotropic accumulation of HS is in-line with the primary neurologic manifestation in MPS III. As mentioned above, HS accumulation causes a modification in the lysosomal environment. The high surplus of undegraded substances can bind to various enzymes, like hydrolases reducing their activity and causing secondary storage of gangliosides and other GAGs. This might be a major contributor to the CNS pathology (Walkley, 2004; Berendsen et al., 2011).

Therefore, a correlation between disease severity and the plasma concentration of HS and urinary total GAGs level has also been studied for MPS III (Ruijter et al., 2013). However, Ruijter et al. (2012) also observed elevated DS levels in in the newborn MPS III dried blood spots. Similar increases have been observed in the liver from MPS III mice (Holley et al., 2018). This accumulation was identified as a result of IDS activity inhibition by MPS III HS (Parker and Bigger, 2018) and is relatively modest compared to HS accumulation.

Throughout the brain of MPS III animal models both microglia and astrocyte activation are present (Villani et al., 2007; Arfi et al., 2011)—similar to MPS I and MPS II. Neuronal loss in MPS III is not consistently observed *via* animal models, but it has been detected in patients with magnetic resonance imaging and autopsy (Sharkia et al., 2014). This may demonstrate the phenotypical variability between human and mice.

The fact, that the CNS has a limited capability of regeneration, a high sensitivity to damage and a necessity of extended cellular survival might explain the severe neural pathology in MPS III patients—concerning both, the CNS and the peripheral nervous system (Benetó et al., 2020).

#### 1.2.2 Inflammatory Immune Response

Secondary storage within neurons has been described by means of a canine model of MPS IIIA. Jolly et al. (2000) interpreted the outcome as accumulation of GAGs and gangliosides. McGlynn et al. (2004) also demonstrated in MPS III animal models, that an accumulation of GM2 and GM3 gangliosides as well as cholesterol play a role in the neuroinflammatory response. Li et al. (2002) demonstrated in a MPS IIIB mouse model important alterations in the expression of several genes involved in HS degradation. The levels of mRNAs for FGF-1 and FGF-2 were lower in the brain regions tested. These alterations may be responsible for the lack of response to acute injury, the insufficiency of neural cell genesis and the capacity for plasticity.

Several other substances, associated with neurodegenerative diseases like Alzheimer or Parkinson disease, have also been shown to accumulate in neurons of MPS III. Increased levels of protein markers, such as lysozyme, hyperphosphorylated tau, phosphorylated tau kinase, GSK3B and amyloid-β are all evident in the brains of MPS III mice (Settembre et al., 2008; Fraldi et al., 2016). Therefore, several experts suggest a possible association between MPS III and neurodegenerative diseases. MPS IIIC mouse brains showed elevations in these markers although there is typically a lower storage than similarly aged MPSIIIA and MPSIIIB mice (Ohmi et al., 2011; Beard et al., 2017). Furthermore, Hamano et al. (2008) showed that α-synuclein aggregation, a specific characteristic for Parkinson’s disease, is present in MPS IIIA and MPS IIIB patients neurons. In MPS IIIA a relation between lysosomal disruption and presynaptic maintenance is assumed to be mediated by a simultaneous loss of α-synuclein and cysteine string protein-α (CSP-α) at nerve terminals. The relative loss of the function of α-synuclein by its abnormal autophagy can be assumed as a major contributor to neuronal degeneration (Sambri et al., 2017). VAMP2, important for neurotransmitter release by docking and fusion of vesicles at the synaptic junction was also shown to be reduced and abnormally distributed in synapses (Wilkinson et al., 2012).

Increased levels of markers for metabolic stress (e.g., glypican) and proteins involved in autophagy (e.g., LC3) have also been studied in MPS mice brains and are much more abundant in MPS III than MPS I or MPS II (Ohmi et al., 2011; Martins et al., 2015). This may contribute to the differences in disease phenotypes (Bigger et al., 2018). Ohmi et al. (2011) suggests that the increased levels of the proteoglycan glypican in MPS III are significant for the brain abnormalities, as glypican is the precursor of the glycan HS and might be metabolized otherwise in MPS III compared to MPS I or MPS II.

Parker and Bigger assume that there is an even more increased production of highly sulfated HS in MPS III, due to an increase of the chain modification enzyme N-Deacetylase/N-Sulfotransferase (NDST). Exocytosed and proteoglycan-bound-HS may interact with TLR4, propagating an inflammatory response. Furthermore, these highly sulfated fragments may be released intracellularly due to lysosomal destabilization and induce the TLR4 pathway and may also directly activate the NLRP3 inflammasome (Parker and Bigger, 2018).

Increased NDST enzyme activity has already been observed by Holley et al. (2011) in MPS I murine brain. Therefore, we hypothesize that levels of this enzyme are higher in MPS III subtypes, subsequently leading to an enhanced TLR4 induction and activation of the NLRP3 inflammasome. Taking into consideration, that IL-1 and cathepsin B expression has shown to be upregulated in brains of MPS III animal models (Arfi et al., 2011). Linking HS, its proteoglycans and fragments to the degree of the neurological disease pathology in MPS.

However, Ausseil et al. (2008) have demonstrated that in MPSIIIB mice deficient TLR4, neurodegeneration can occur autonomously of microglial activation by HS, assuming that inflammation *via* this pathway is not the main reason for pathology in MPS III. The subsequent discovery of the inflammasome and demonstration that alternate substrates and pathologies may also eventually lead to inflammasome activation (Parker et al., 2020) with elevated levels of pro-inflammatory cytokines, such as IL-1, TNF-α, MCP-1, and MIP-1α, all provide support for this (Ausseil et al., 2008; Arfi et al., 2011; Wilkinson et al., 2012). A recent murine model of MPS IIIA described that a restoration of the lysosomal pathway was associated with reduced neuroinflammation and improvement of cognitive decline (Monaco et al., 2020).

Furthermore, it has been proven, that abnormal autophagy is one of the key drivers regarding the inflammatory immune response of MPS IIII. Original studies on the impairment of autophagy in LSDs were performed in a MPS IIIA mouse model inter alia. The disruption of the autophagic pathway was reported by Settembre et al. (2008) and might be linked to an inefficient degradation of exogenous aggregate-prone proteins. Consequently, this results in massive accumulation of dysfunctional mitochondria, as well as polyubiquitinated proteins. The role of autophagy in the pathophysiology of the disease, in connection with phenotypical appearance has been further studied by Webber et al. (2018) by means of an MPS IIIA *Drosophila* model. These MPS IIIA flies showed a progressive defect in climbing ability—a hallmark of neurological dysfunction. Autophagy-related proteins (Atg1 and Atg18), superoxide dismutase enzymes (Sod1 and Sod2), as well as heat shock protein (HSPA1) have been identified *via* genetic screen as prominent factors for modifying the climbing phenotype. Moreover, a decreasing HS biosynthesis significantly worsens the behavioral phenotype.

Abnormal mitochondrial numbers and morphology have been observed in MPS IIIC mice and could subsequently lead to oxidative stress (Martins et al., 2015). Pathological findings have been described in detail in an animal model of MPS IIIC by Pshezhetsky (2016). The progressive accumulation of pleomorphic, swollen mitochondria containing disorganized or reduced cristae has been reported as one of the most prominent pathological changes in MPS III neurons. This finding has been observed in all parts of the brain. Neurons containing swollen mitochondria are present as early as at 5 months of age and by the age of 12 months the mitochondrial dysfunction can be identified in the major part of neurons. The author speculates that cytokines have the ability to cause mitochondrial impairment, due to the release of ROS/RNS and oxidative stress eventually leading to neuroinflammation and cell death (Pshezhetsky, 2016).

While some experts suggest that oxidative stress may play an important role in early stages of disease (Villani et al., 2009; Villani et al., 2012), others assume that oxidative stress is not a consequence, but rather a cause of neuroinflammation, as oxidative stress is present at an early stage in the human brain (Trudel et al., 2015).

In 2017 Roca et al. (2017) generated for the first time a MPS IIID animal model, due to the fact that the subtypes D is the most infrequent one of these four. However, each mouse model of MPSIIIA-D demonstrated variations in the time at onset and severity of the disease pathology, such as GAG build-up, degree of lysosomal distention, severity of neuroinflammation, onset of behavioral abnormalities and lifespan.

As mentioned above, several experts hypothesize that behavioral differences might be due to the localization and or to the amount of GAG storage, as well as variances in GAG chain length, sulfation patterning or chemical modifications at the non-reducing terminus of the partially degraded HS (Węgrzyn et al., 2010; Wilkinson et al., 2012; Bigger et al., 2018). Therefore, the N-sulfate (MPS IIIA), amino (MPS IIIC) or N-acetyl (MPS IIIB and MPS IIID) moieties may be the decisive factors for phenotypical presentation and severity of neurological symptoms in MPS III.

### 1.3 Morquio Syndrome (MPS IV)

#### 1.3.1 Metabolic Dysfunction

MPS IV is caused by a deficiency of the enzymes galactosamine-6-sulfatase (MPS IVA) or β-galactosidase (MPS IVB). The deficiency of the respective enzyme leads to the accumulation of KS and/or C6S in lysosomes and ECM (Muenzer, 2011). In MPSIV, skeleton is severely affected, liver and spleen might be slight enlarged, while the CNS is spared.

The prominent role of KS fulfils a number of specific biological functions, including tissue hydration, cellular recognition of protein ligands, axonal guidance, cell motility, and embryo implantation (Weyers et al., 2013). Shimada et al. (2015) demonstrated, that di-sulfated KS is supposed to be a novel biomarker for MPS IV, however di-sulfated KS levels are higher in MPS IVA than in MPS IVB. Compared with MPS IVA patients, the patients with MPS IVB usually show a milder skeletal dysplasia phenotype.

KS level in MPS IVA patients varies with age and clinical severity (Tomatsu et al., 2004). Blood KS levels in patients with severe MPS IVA are higher than in patients with the attenuated form. Both blood and urine KS are reliable biomarkers in younger patients. However, the synthesis of KS decreases after adolescence and KS levels in MPS IVA patients are naturally normalized or near normalized by the age of 20 years, as the growth plate is closed or damaged (Tomatsu et al., 2015; Khan et al., 2017).

Besides the storage of KS, there is also an accumulation of another GAG, called C6S, documented in MPS IVA patients. C6S accumulates in heart valves and aorta and related foam cells/macrophages contain C6S rather than KS (Yasuda et al., 2013). The KS accumulation is assumed as the primary driver for bone dysplasia. The role of C6S in MPS IVA is still uncertain (Khan et al., 2017).

Nonetheless, the study by Tan and Tabata (2014) came to the conclusion that C6S attenuates the inflammatory response in murine model *via* a significantly reduced production of IL-6 and TNF-α. Therefore, a more detailed analysis of tissue distribution pattern of C6S is urgently needed to reveal pathogenic roles of this GAG in MPS IVA. C6S may play a considerable part in the biochemical pathology or even contribute the intensification of the inflammatory effects, leading to skeletal abnormalities and short stature, triggered by KS.

#### 1.3.2 Inflammatory Immune Response

Several MPS IVA murine models have already been studied (Tomatsu, 2003; Tomatsu et al., 2005; Tomatsu et al., 2007). Overall, none of these model mice have the same phenotypical feature seen in human patients, even though abundant storage materials do accumulate in multiple tissues. The major reason may be that rodents, including mice, synthesize far less KS compared to human—up to 100 folds lower (Khan et al., 2017).

Oxidative stress may be a hallmark in MPS IV, according to results by Donida et al. (2015) and Fujitsuka et al. (2019). Their results showed high lipid and protein oxidative impairement, reduced antioxidant defenses and elevated levels of inflammatory markers.

Pro-inflammatory cytokines as well as GAG levels and oxidative stress parameters have been analyzed by Donida et al. (2015) in urine and blood samples from MPS IVA patients under ERT and in healthy matched controls. Patients affected by MPS IVA demonstrated decreased antioxidant defense levels, evaluated *via* glutathione content and superoxide dismutase activity. The damage of lipids and proteins has been evaluated *via* urine isoprostanes and di-tyrosine levels and plasma sulfhydryl groups. MPS IVA patients compared to controls presented a higher DNA damage with an origin in pyrimidine and purine bases. Furthermore, the pro-inflammatory cytokine IL-6 was increased in MPS IVA patients and presented an inverse correlation with glutathione levels—consistent with studies involving animal models of MPS I and MPS III (Ohmi et al., 2003; Arfi et al., 2011).

Fujitsuka et al. evaluated the levels of 8 pro-inflammatory factors (EGF, IL-1β, IL-6, MIP-1α, TNF-α, MMP-1, MMP-2, and MMP-9), collagen type II, and DS, HS (HS0S, HSNS), and KS (mono-sulfated, di-sulfated) in blood samples of MPS II, MPS IVA and MPSIVB patients. Eight biomarkers were significantly elevated in untreated MPS IVA patients as well: EGF, IL-1β, IL-6, MIP-1α, MMP-9, HSNS, mono-sulfated KS, and di-sulfated KS, and four biomarkers were elevated in MPS IVA patients under ERT: IL-6, TNF-α, mono-sulfated KS, and di-sulfated KS. Two biomarkers were significantly elevated in untreated MPS IVB patients: IL-6 and TNF-α. Reversely, collagen type II levels were significantly reduced in untreated and ERT-treated MPS II patients and untreated MPS IVA patients (Fujitsuka et al., 2019). Previous animal models have shown that enhanced apoptosis of MPS chondrocytes leads to a reduction of proteoglycans and total collagen in the cartilage (Simonaro et al., 2001). It is essential to identify if the increase of collagen type II correlates with specific clinical improvements, like skeletal dysplasia.

Overall, three pro-inflammatory factors (IL-6, TNF-α, and MMP-1) showed significantly different levels in untreated MPS IVA patients compared to ERT treated MPS IVA patients. However, there was no decrease of KS in the ERT-treated group (Fujitsuka et al., 2019). This striking discrepancy between biomarkers and blood KS level cannot declared by the currently limited published data.

These data presented suggest that an inflammatory immune response, leading to skeletal abnormalities, may occur in MPS IV due to impaired autophagy and the subsequent mitochondrial damage. The data presented showed a possible relation between inflammation and oxidative stress in MPS IV disease (even under ERT in MPS IVA).

Secondary storage as well as membrane permeabilization has so far not been studied, but should not be ruled out. An induction of TLR4 is unlikely due to the structure of the respective GAGs (KS and C6S). Indeed C6S may even reduce inflammation *via* suppression of NF-κB translocation (Tan and Tabata, 2014).

### 1.4 Maroteaux-Lamy Syndrome (VI)

#### 1.4.1 Metabolic Dysfunction

MPS VI is caused by a deficiency of the enzyme N-acetylgalactosamine-4-sulfatase (ARSB) leading to an accumulation of DS in lysosomes and ECM (Muenzer, 2011). Due to the fact, that an accumulation of the same GAGs as in MPS I and MPS II is present, but without a storage of HS, the cytopathology and appearance of clinical manifestations is quite similar, though without any neurological involvement. As a consequence of DS accumulation, cartilage apoptosis, synovial hyperplasia, recruitment of macrophages, transformed connective tissue matrices and inflammatory joint destruction have been identified.

#### 1.4.2 Inflammatory Immune Response

Secondary storage has been described by a feline model of MPS VI. Pyramidal neurons were demonstrated to contain abnormal quantities of GM2 and GM3 gangliosides and unesterified cholesterol. Some animals evaluated in this study also received HSCT, but no variations in neuronal storage were reported between treated and untreated animals. The study demonstrated that deficiency of ARSB activity may cause a metabolic abnormality in neurons of CNS in cats. These changes cannot be straightforwardly corrected by HSCT. Given the close pathological and biochemical similarities between feline and human, it is conceivable that MPS VI patients have similar neuronal involvement (Walkley, 2004). An accumulation of gangliosides may trigger changes in dendrite and axon morphology. This is supposed to cause synaptic dysfunction, neuronal cell death and neurodegeneration in MPS VI patients brain (Bigger et al., 2018).

In DS storage diseases such as MPS VI, there is no evidence of CNS involvement, although MPS VI is characterized by similar secondary storage, pathology and inflammation (Bigger et al., 2018). However, it should be pointed out that structural brain abnormalities have also been reported for MPS types that are not typically associated with neurocognitive impairment. For example, Azevedo et al. (2013) described in patients affected by MPS VI enlarged perivascular spaces and white matter lesions, without IQ correlation.

The important role of TLR4 signaling in MPS bone and joint disease has been revealed by Simonaro and colleagues by an MPS VI mouse model. An elevation in the expression of genes encoding TLR4 (e.g., LBP, MyD88, CXCR4, and MIP-1a) and matrix metalloproteinase (e.g., MMP-1, MMP-2, MMP-9, and MMP-13) was found in synovial cells and cartilage. An elevated expression of the CD44 adhesion receptor was detected in fibroblasts and chondrocytes of MPS VI rats—adequate for the TLR4 activation (Simonaro et al., 2008; Simonaro et al., 2010). This work suggests, that an inflammatory immune response *via* TLR4 induction can also be triggered by DS.

However, we assume, that the increased production and release of inflammatory markers is mainly initiated by mitochondrial dysfunction. The structure of DS could be taken to imply that an activation of TLR4 is hardly possible.

Tessitore et al. (2009) studied several anomalies along with inflammation, in association with DS storage in MPS IV and assume abnormal autophagy as key driver. Mitochondrial dysfunction in fibroblasts from MPS VI patients with an accumulation of polyubiquitinated proteins and overproduction of ROS/RNS, due to impaired autophagy has been observed. Furthermore, they showed similar anomalies, along with inflammation and apoptosis, in association with DS accumulation in the visceral organs of MPS VI rats, but not in their CNS where DS storage is absent. They assume that DS storage disrupts the capability of lysosomes in order to completely and correctly degrade substances from the autophagic pathway, consequently leading to cell toxicity.

### 1.5 Sly Syndrome (MPS VII)

#### 1.5.1 Metabolic Dysfunction

MPS VII is caused by a deficiency of the enzyme β-glucuronidase, leading to an accumulation of several GAGs in lysosomes and ECM (Muenzer, 2011). HS and DS are the main GAG accumulating in MPS VII, a storage of C4S and C6S has also been proven.

As already pointed out above, DS accumulation is characteristically associated with somatic and skeletal involvement in MPS I, MPS II, MPS VI, and MPS VII, while HS storage may be the key driver for neurodegeneration in MPS I, MPS II, MPS III, and MPS VII. The storage of CS derivatives (here C4S and C6S) is linked to skeletal abnormalities and short stature, an accumulation of C6S has also been mentioned in MPS IVA.

Depending on whether there is a complete absence or higher levels of the lysosomal enzyme MPS VII can present as severe or mild with or without neurological involvement. Furthermore, nearly half of all reported cases had non-immune hydrops fetalis *in utero* or at delivery (Holtz et al., 2020).

#### 1.5.2 Inflammatory Immune Response

Simonaro et al. (2010) have also proven an activation of the TLR4 pathway in MPS VII. Interestingly, an inactivation of TLR4 in MPS VII mice had a significant positive effect on their growth, greater length of the bone, unchanged bone density, improved growth plates, and normalization of TNF-α level. Furthermore the author assumed, that peripheral inflammation may largely be driven and controlled by TNF-α (Simonaro et al., 2005; Simonaro et al., 2008; Simonaro et al., 2010). However, Parker and Bigger put forward the hypothesis that this is not the case in the brain, where TNF-α might be secondary to other pro-inflammatory factors, such as IL-1. Nevertheless, it is unquestionable that peripheral inflammation has the ability to influence central events (Parker and Bigger, 2018).

Inflammatory immune responses have also been proven *via* gene expression profile analysis in brains from MPS VII mice. Genes related to the immune system and inflammation were upregulated, while major oligodendrocyte genes were downregulated. Specific brain areas may be more vulnerable to inflammation than others, as patterns of gene expression dysregulation appeared specific for different brain regions (Parente et al., 2016).

Walton and Wolfe demonstrated similar neural results by a canine MPS VII model, as Li et al. (2002) *via* MPS IIIB mouse model. Neuronal cells mature less than normal ones, although these differences were only present an early phase after isolation. This study was carried out with neural progenitor cells, since they have a prominent role for brain development and function (Walton and Wolfe, 2007).

Studies completed in a MPS VII canine model showed an impaired secondary ossification initiation phase and a possible dysregulation of signaling pathways modulating bone development and bone ossification. An aberrant persistence of Sox9 protein was detected in chondrocytes and these cells were incapable to properly transit from proliferation to hypertrophy (Oguma et al., 2007). Another recently conducted work by Peck et al. (2019) has been focused on signaling pathways essential in the regulation of endochondral ossification. The osteoactivin gene was highly upregulated in MPS VII. However, key factors of the osteogenic pathways, like Wnt/β-catenin or BMP signaling were unaltered, suggesting that important bone formation pathways are not activated.

The osteopenia described in MPS VI and MPS VII is possible due to early chondrocyte death and decreased activity of osteoblasts. This could result in incomplete and disorganization of the growth plate (Opoka-Winiarska et al., 2013). This has so far not been described in MPS I and MPS II.

Bartolomeo et al. (2017) demonstrated, that an impaired autophagy may also be present in MPS VII. Their murine model has proven that deregulation of mTORC1 signaling adversely affects bone growth in LSDs. Lysosomal dysfunction induces a significant mTORC1 activation in chondrocytes. Consequently, chondrocytes are unable to successfully secret collagens. The autophagic rescue resulted in restored levels of collagen in the cartilage and improvements of the bone phenotype.

### 1.6 Natowicz Syndrome (MPS IX)

#### 1.6.1 Metabolic Dysfunction

MPS IX is caused by a deficiency of the enzyme hyaluronidase, leading to an accumulation of hyaluronan (HA) in lysosomes and ECM (Muenzer, 2011). HA is defined as GAG, although it differs from other GAG members as it is not sulfated or protein-linked. Furthermore, HA is synthesized at the cell membrane and not in the Golgi apparatus. HA is an important component of the ECM of connecting tissues (Triggs-Raine, 2015).

MPS XI research has been conducted to a lesser extent, due to the fact, that this MPS type is the rarest of all. Therefore, neither the biochemical pathology nor the inflammatory immune response (see below) is fully understood.

#### 1.6.2 Inflammatory Immune Response

Polydisperse HA fragments, with an average molecular weight of 200 kDa, have been described to stimulate chemokines, cytokines, growth factors, proteases and ROS/RNS by macrophages. HA fragments include various inflammatory effects, such as activation of macrophages and dendritic cells as well as stimulating the transcription of inflammation-related genes, including TNF-α, IL-12, IL-1β, and MMPs (Termeer et al., 2000).

Multiple studies have shown that HA fragments can also be protective, although studies regarding low-molecular-weight HA-fragments initially illustrated a pro-inflammatory response. The strongest TLR4 induction was observed with HA of 35 kDa (Hill et al., 2012). Simonaro et al. (2010) also assumed that an activation of the TLR4 pathway may be linked to HA.

Martin et al. (2008) characterized a Hyal1 null mouse model of MPS IX and compared the phenotype with the human disease. An increased number of chondrocytes displaying intense HA staining in the epiphyseal and articular cartilage of Hyal1 null mice, demonstrating an HA storage. Increased levels of HA have not been detected—neither in the serum nor in the non-skeletal tissues. This finding indicates that osteoarthritis is the key clinical feature in MPS IX.

An overview of all MPS types and subtypes is given in Table 1 below.

**TABLE 1:** MPS classification.

Type	Eponym	Enzyme (deficit)	GAG (storage)	Clinical features	Gen (−locus)	ERT/HSCT	Incidence per 100,000 live births
MPS I	Hurler (severe)	α-L-iduronidase	DS, HS	Coarse facial features, short stature, cognitive decline, skeletal abnormalities (=dystosis multiplex) corneal opacity, cardio respiratory disease, hepatosplenomegaly frequent airway and ear infections	IDUA (4p16.3)	✓✓ Laronidase, Aldurazyme^®^	0.69–1.66
	Hurler-Scheie (intermediate)			Coarse facial features short stature moderate cognitive decline cardio respiratory disease, hepatosplenomegaly, corneal opacity, skeletal abnormalities (=dystosis multiplex) frequent airway and ear infections			
	Scheie (mild)			Coarse facial features, no cognitive decline, cardio respiratory disease, skeletal abnormalities (=dystosis multiplex) hepatosplenomegaly, corneal opacity frequent airway and ear infections			
MPS II	Hunter (severe/mild)	Iduronate-2-sulfatase	DS, HS	Coarse facial features, short stature, skeletal abnormalities (=dystosis multiplex), cardio respiratory disease, hepatosplenomegaly, (cognitive decline) Hearing loss frequent airway and ear infections	IDS (Xq28)	✓(✓) Idursulfase, Elaprase^®^	0.30–0.71
MPS III	Sanfilippo A	N-sulfoglucosamine sulfohydrolase	HS	Cognitive decline, hearing loss mild skeletal abnormalities (=dysostosis multiplex) hepatosplenomegaly	SGSH (17q25.3)	×	0.29–1.89
	Sanfilippo B	α-N-acetylglucos-aminidase			NAGLU (17q21.2)		0.42–0.72
	Sanfilippo C	Heperan-α-glucosaminide-N acetyltransferase			HGSNAT (8p11.21)		0.07–0.21
	Sanfilippo D	N-acetylglucosamine 6-sulfatase			GNS (12q14.3)		0.1
MPS IV	Morquio A	Galactosamine-6-sulfatase	KS, C6S	Skeletal abnormalities (=dysostosis multiplex), short stature frequent airway and ear infections	GALNS (16q24.3)	✓(✓) Elosulfase-α,Vimizim^®^	0.22–1.3
	Morquio B	β-galactosidase	KS		GLB1 (3p22.3)	×	0.02–0.14
MPS VI	Maroteaux-Lamy (severe/mild)	N-acetylgalactos-amine-4-sulfatase	DS	Coarse facial features, short stature skeletal abnormalities (=dysostosis multiplex), corneal opacity cardio respiratory disease, hepatosplenomegaly frequent airway and ear infections	ARSB (5q14.1)	✓ Galsulfase, Naglazyme^®^	0.36–1.3
MPS VII	Sly (hydrops fetalis/severe/mild)	β-glucuronidase	DS, HS, C4S, C6S	Coarse facial features, short stature, cardio respiratory disease, skeletal abnormalities (=dystosis multiplex), corneal opacity, (cognitive decline)	GUSB (7q11.21)	✓(✓) Vestronidase- α, Mepsevii^®^	0.05–0.29
MPS IX	Natowicz	Hyaluronidase	HA	Short stature, frequent ear infections	HYAL1 (3p21.31)	×	4 case reported

## 2 Repurposing: Immunomodulatory Molecules

The observation that substantial inflammation induced cytopathology takes place in progressive manner, even in patients under ERT, directs us towards a role of inflammation as a key contributor to the unmet clinical need in MPS. In the following section, we summarize the available evidence on immunomodulation in MPS and give an overview on ongoing (pre-) clinical trials with immunomodulatory molecules utilizing this treatment strategy.

Currently there are different types of immunomodulatory molecules under investigation.

The characterization of cellular inflammatory processes, which can trigger the pathophysiology of MPS, is now providing evidence to address the limitations of ERT and HSCT and to identify novel therapeutic targets. Figure 1, aforementioned, shows the inflammatory immune response with several promising target points to intervene.

Due to the current knowledge on inflammation, these processes seem feasible to intervene in the activation of the innate immune response:

(1) TLR4 induced inflammation, with TNF-α as key driver.
(2) Activation of the NLRP3 inflammasome, with IL-1β as key driver.
(3) Perturbed autophagy and secondary mitochondriopathy.
(4) Increased oxidative stress.
(5) Impaired Ca^2+^ homeostasis and signaling.
(6) Increased lysosomal membrane permeability with release of cysteine proteases.

**FIGURE 2:**
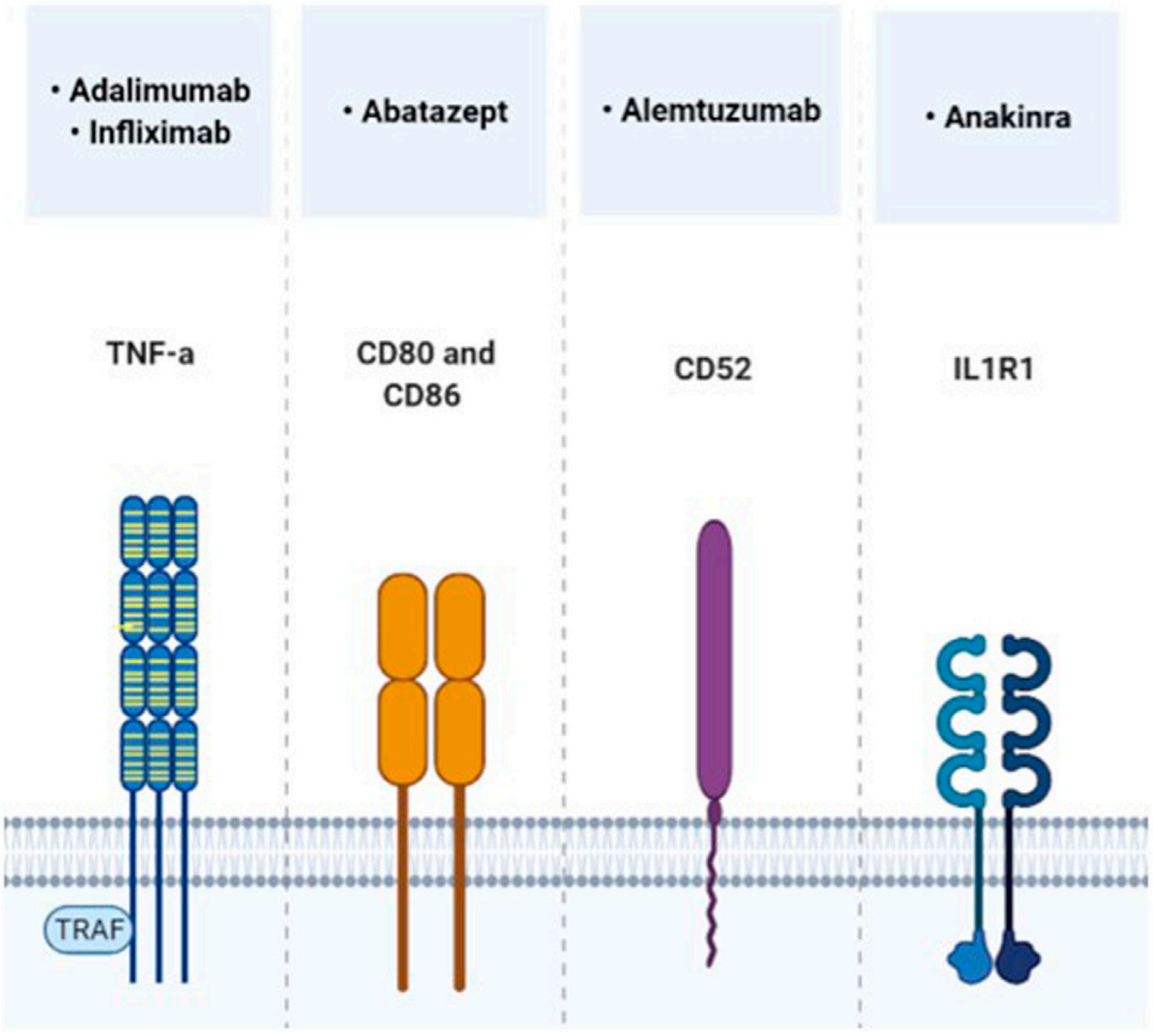
Direct immunomodulatory molecules, which target the cytokine receptor pathway and have already been tested in MPS trials.

**FIGURE 3:**
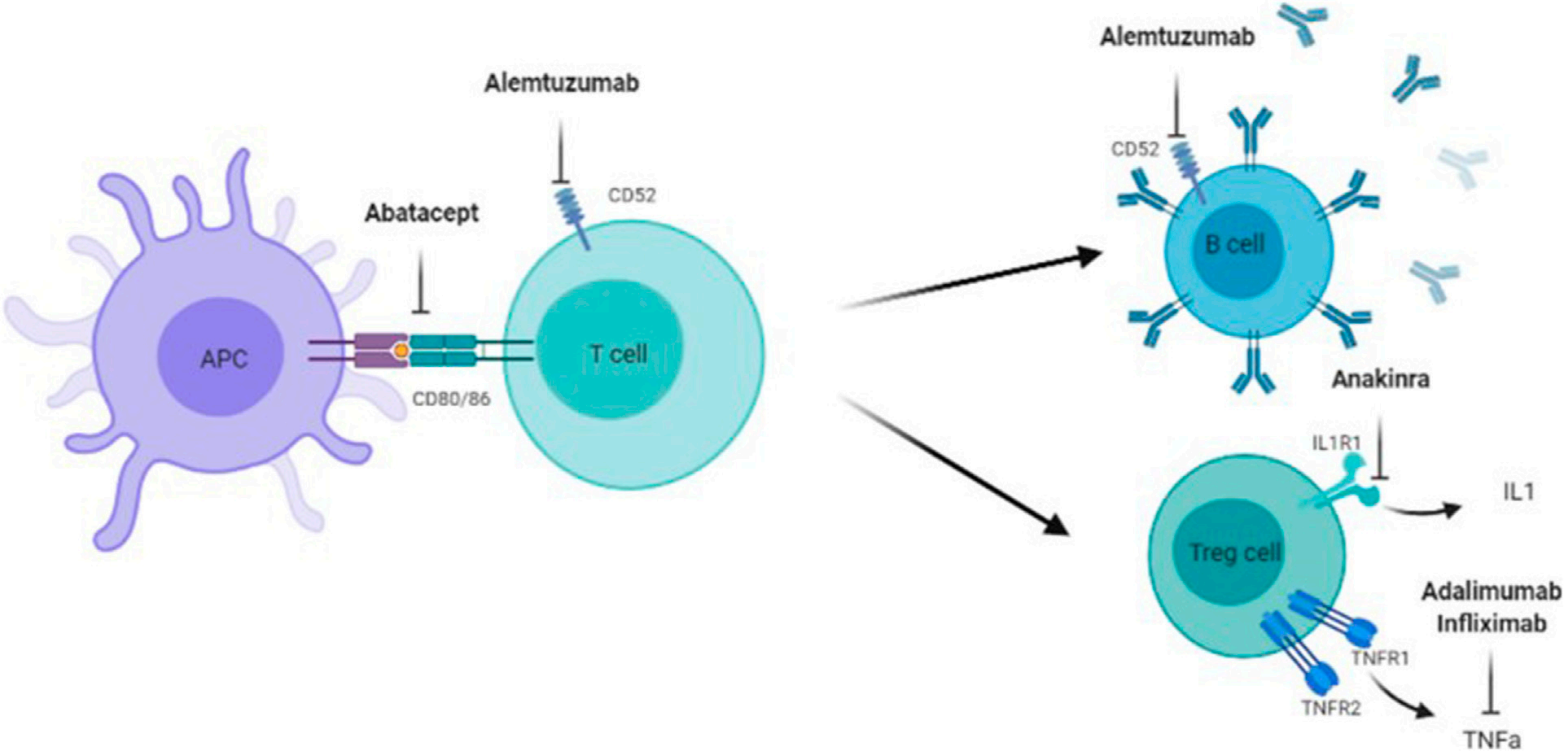
Target points of Abatacept, Alemtuzumab, Anakinra, Adalimumab, and Infliximad. Adaliminab and infliximab block the action of INF-a, Anakinra inhibits the ILI receptor and a following ILI release, Alemtuzumab targets mature B- and T-cells *via* CD52, Abatacept prevents full T-cell activation *via* CD80 and CD86 between antigen presenting cell (APC) and T-cell.

In the following, the focus is set on relevant target points that can be addressed by approved immunomodulatory drugs, which have already been studied in MPS in preclinical or clinical setting (Figures 2, 3). Evidence supports a therapy with biologicals and antioxidants. Perhaps several different targets have to be addressed simultaneously to receive a beneficial effect, especially in the CNS. To date treatment combinations have only been studied in MPS with ERT.

### 2.1 TLR4 Induced Inflammation, With TNF-α as Key Driver

Promising treatment strategies targeting TLR4 pathways include biologicals as well as Pentosan Poylsulfate (PPS). These directly target the transcription and release of pro-inflammatory regulators. Furthermore, TAK242 a TLR4 inhibitor is currently under investigation by Takeda (Rice et al., 2010).

#### 2.1.1 Adalimumab

Adalimumab, a human monoclonal antibody that inhibits TNF-α, has been proven effective and safe by Polgreen et al. (2017) for patients with MPS I and II in a 32-week, randomized, double blind, placebo-controlled, crossover study (NCT02437253; NCT03153319). Two patients, one with MPS I and one with MPS II, completed the clinical trial. There were no serious adverse events reported. Data from this small pilot study suggest that Adalimumab may improve pain, physical and neurogenic function in children with MPS I or II. This trial was based on encouraging results by Simonaro et al. (2010) and Eliyahu et al. (2011). Both evaluated the efficiency of another TNF-α inhibitor, Infliximab, based on an MPS animal model. Their work has underscored the importance of the TLR4/TNF-α inflammatory pathway in the skeletal pathology of MPS. Moreover, they demonstrated that combining ERT with anti-TNF-α drugs improves the outcome. They further validated the use of TNF-α and other immunomodulatory molecules as potential biomarkers for MPS.

#### 2.1.2 Abatacept

Abatacept, a disease modifying anti-rheumatic drug, blocks the co-stimulatory signal mediated by CD28^−^CD80/86 engagement. This pairing is required for T-cell activation. A single arm phase I study on Abatacept is currently running (NCT01917708). This substance is combined with cyclosporine and mycophenolate mofetil as graft versus host disease prophylaxis in 10 children undergoing unrelated HSCT for serious non-malignant diseases. Patients affected by MPS I may also be enrolled. Participants receive 4 doses of Abatacept 10 mg/kg/dose i.v. (days −1, +5, +14, +28) and are followed for 2 years. This trial aims to assess the tolerability and the immunological effects of Abatacept. First results may be posted in the year 2021, as the actual study completion data is given as 19th September 2019.

#### 2.1.3 Alemtuzumab

Alemtuzumab is a monoclonal antibody used as immune reconstitution therapy for patients with aggressive multiple sclerosis (MS). Alemtuzumab targets CD52 leading to profound but transient peripheral immunodepletion (Moser et al., 2020). Alemtuzumab has been tried in phase II studies in MPS patients (type I and/or type II and/or type III and/or type VI). Alemtuzumab was used after HSCT and combined with other interventions (e.g., Busulfan) as graft versus host prophylaxis. Three of a total of 44 patients enrolled were affected by MPS. The MPS patients consisted of one with Hurler syndrome, who underwent transplant at 22 months of age and two children with Hunter syndrome who underwent transplant at 10 months and 1.25 years of age, respectively. They continued to achieve and improve skills, but showed mild developmental delay (Vander Lugt et al., 2020).

The high molecular weight (MW) of monoclonal antibodies indicates a low CNS bioavailability, but analog to MS, effects on the peripheral immune system might secondarily impact CNS inflammation. In addition, Torres-Acosta et al. (2020) observed CNS effects induced by systemic TNF-α lowering in a mouse model on Alzheimer’s disease. Thus indicating promising beneficial effects in neuronopathic MPS patients. The ability to lower peripheral inflammatory responses *via* antibodies may also impact directly on the CNS, as most cytokines (the effectors of the immune system) are produced at greater volume in the periphery rather than the brain, but cytokines can cross the blood brain barrier and exert their effects in both compartments.

#### 2.1.4 Pentosan Polysulfate

Pentosan Polysulfate (PPS), an anti-inflammatory and pro-chondrogenic molecule, has shown to supress the activation of TLR4 in MPS in preclinical and clinical settings. PPS has been tested *in vitro* and *in vivo* studies. Early s.c., PPS therapy presented a decrease in neuroinflammation and—degeneration in a MPS IIIA mouse brain (Guo et al., 2019). Injected PPS shows an enhanced delivery to tissues like bone and cartilage, than orally administered PPS and has shown beneficial aspects in an osteoarthritis MPS VI mouse model (Frohbergh et al., 2014). Simonaro et al. (2016) observed PPS to decrease pro-inflammatory markers and GAG accumulation in the urine and importantly, a significant cytokine reduction in the CSF of MPS I dogs.

An open label, randomized, monocentric phase II study administering s.c., PPS in a dose of 1 or 2 mg/kg in four MPS I patients. PPS was injected weekly for 12 weeks, afterwards biweekly for 12 weeks. The treatment was well tolerated and the results showed a significant decrease of urinary GAG excretion and an improvement of the skeletal pathology—in both, the 1 and 2 mg/kg group. In patients with mild pain, the pain intensity remained stable, however in those with severe pain a significant amelioration has been monitored (Hennermann et al., 2016). In a pilot clinical safety study three male adults suffering from MPS II received weekly PPS injections for 12 weeks. Results showed decreases in the inflammatory cytokines TNF-α and macrophage migration inhibitory factor (MIF) (Orii et al., 2019). Nevertheless, the exact inflammatory mechanism by which PPS affects GAG storage and stimulates chondrocyte formation is yet unclear (Schuchman et al., 2013; Simonaro et al., 2016).

### 2.2 Activation of the NLRP3 Inflammasome, With IL-1β as Key Driver

#### 2.2.1 Anakinra

The induction of TLR4 has been linked to the production and release of IL-1β, as well as the relationship between autophagy and inflammation by the inflammasome (Parker and Bigger, 2018).

IL-1β is a key driver regarding the activation and upregulation of the immune system. On this account, a further immunomodulatory drug is currently under investigation, with focus on chronic brain inflammation.

Anakinra, a human IL-1R antagonist, is presently being investigated in an open-label, single center, pilot study of 20 MPS III patients, aged ≥4 years (NCT04018755). After an initial 8 week-observational period, patients receive 100 mg s.c., of Anakinra for 36 weeks. The endpoints include, but are not limited to, changes in behavior, sleep, stooling, communication, mood, and gait; Other key outcome parameters addressed are seizure frequency, disordered movement and fatigue.

Brain exposure of systematically dosed biologics is naturally challenging to predict in both clinical and pre-clinical settings. One reason is, that the composition of the CSF does not entirely reflect the contents of the brain interstitium and reverse. Moreover, samples of the brain interstitial fluid are not easily available. Large proteins, like monoclonal antibodies are estimated to enter the brain compartment in a range of 0.1%–1% only. Sjöström et al. (2021) recently reported, that Anakinra passes the human brain-like endothelial monolayer at 4.7-fold higher rate than the monoclonal antibodies tested. Therefore, Anakinra may reach the brain compartment at clinically relevant levels, encouraging the utility of Anakinra for treatment of neuroinflammatory diseases.

### 2.3 Perturbed Autophagy and Secondary Mitochondriopathy

Several studies performed in animal models have linked impaired autophagy and oxidative stress to MPS. Therefore, rescuing the autophagic flux and mitochondrial dysfunction may be beneficial for the unmet medical need MPS patients, as described in several animal models. Altered autophagy has been reported in MPS II (Fiorenza et al., 2018), MPS IIIA (Webber et al., 2018), MPS IIIC (Pshezhetsky, 2016), MPS VI (Tessitore et al., 2009) and MPS VII (Bartolomeo et al., 2017).

It has already been demonstrated, that an inhibition of autophagy can improve the efficacy of several cancer therapies—preclinical evidence is growing. To date, hydroxychloroquine (HCQ) is the only approved drug, which targets autophagy. However, studies of pharmacodynamics reported that high doses of HCQ (up to 1,200 mg/day) produce only modest inhibition *in vivo* and might be inconsistent (Shi et al., 2017). Furthermore, HCQ fails to inhibit autophagy in an acid milieu, as the cellular uptake of HCQ is significantly reduced. The inhibitory interaction of HCQ with the lysosome might be contributing to the lysosomal disruption in MPS and beyond. However, preclinical studies are still lacking and due to the potency of the drug other autophagy inhibitors are needed. To date, multiple molecules are in the pre-clinical stage of investigation (e.g., 3-Methyladeninin, Wortmannin, LY294002, SBI-0206965, Spautin-1, SAR405, NSC185058, Verteporfin, Lys05, ROC325, and Spautin-1) (Chude and Amaravadi, 2017).

### 2.4 Increased Oxidative Stress

Targeting the increased oxidative stress, consequences of defects in mitophagy and mitochondrial dysfunction, may also be a promising treatment option. Oxidative imbalance has been described even in the early stages of the disease course and studied in MPS IIIA (Arfi et al., 2011), MPS IIIB (Trudel et al., 2015), MPS IVA (Donida et al., 2015) animal models and MPS I (Pereira et al., 2008) and MPS II (Filippon et al., 2011) blood samples.

Novel therapeutic strategies for MPS should not only be focused on lowering the GAG level, but also on ameliorating the whole spectrum of cellular process, causing reduction of symptoms. Antioxidant therapy may have a major impact of disease progression. Although these approaches are not expected to be curative, they may help in improving quality of life. Resveratrol and Coenzyme Q10 are promising treatment options, which have already been tested in a preclinical MPS setting.

#### 2.4.1 Resveratrol

Resveratrol, a natural phenol and phytoalexin appears as a potential candidate, as its mechanism of action in autophagy stimulation is pleiotropic. Antioxidant, anti-inflammatory and autophagy-modulating properties of Resveratrol are widely reported (Rintz et al., 2020). The only research published so far with the use of Resveratrol in MPS has been conducted with a *Drosophila melanogaster* model. This MPS VII animal model has demonstrated that Resveratrol treatment improved behavior and crossed the BBB in flies (Bar et al., 2018). Resveratrol has been verified safe and well tolerated in various animal and human studies, including neurodegenerative disorders (e.g., Alzheimer disease, Parkinson disease). Potential adverse events are considered as rather unlikely (Rintz et al., 2020).

#### 2.4.2 Coenzyme Q10

Coenzyme Q10 (CoQ10) levels are reduced in patients suffering from MPS III. Cultured fibroblasts from MPS IIIA and B have been treated with 50 μmol/L CoQ10 and a mixture of antioxidants. Increased enzymatic activity was especially observed in MPS IIIB cell lines and decreased GAG storage was noticed in some MPS IIIA and MPSIIIB fibroblasts, especially the ones presenting enhanced exocytosis (Matalonga et al., 2014). A nutritional study in nine MPS III patients explored the nutritional status by analyzing various vitamins and micronutrients in blood and in CSF. CoQ10 plasma concentrations were significantly deficient in 8 of 9 participants (Yubero et al., 2016).

### 2.5 Impaired Ca^2+^ Homeostasis and Signaling

To date there is no effective therapy concerning membrane permeabilization and the respective release of cysteine proteases.

Caspase antagonists, such as Balnacasan (VX765) targeting Caspase 1 and Pralnacasan (VX-740) targeting IL-1β -converting-enzyme (ICE), inhibit the release of LPS induced IL-1β and IL-18 (Xu et al., 2019; Flores et al., 2020; Hook et al., 2020). Balnacasan has already proven to be safe for several medical applications, including treatment of epilepsy, arthritis, heart attack, but is less effective and therefore not yet approved. A Cathepsin B inhibitor (CA074) is currently under investigation for several diseases—from mercury induced autoimmunity to cancer. A subsequent attenuation of the systemic adaptive immune response has been reported in preclinical studies (Toomey et al., 2014).

Furthermore, there are several chemicals, which are capable to inhibit cysteine proteinases, like iodacetamine. Nevertheless, none of these are considered to be safe enough as pharmaceutical for human usage.

Table 2 summarizes immunomodulatory therapies, which are already under investigation in MPS.

**TABLE 2:** Preclinical- and clinical development status of innovative immunomodulatory therapies that have been tested in MPS (NCT clinicaltrials.gov identifier number).

MPS type	Preclinical study *in vitro*	Preclinical study animal model	Clinical trial (completed/ recruiting/active)	Reference
Biologicals				
Adalimumab				
MPS I			Phase I and II: NCT02437253; NCT03153319	Polgreen et al. (2017)
MPS II			Phase I and II: NCT02437253; NCT03153319	Polgreen et al. (2017)
Infliximab				
MPS VI		Infliximab in MPS VI mice		Simonaro et al. (2010), Eliyahu et al. (2011)
Abatacept				
MPS I			Phase I: NCT01917708	
Alemtuzumab				
MPS I			Phase II: NCT01962415 (terminated) Phase II: NCT00668564 Phase II: NCT01043640	Miller et al. (2011), Vander Lugt et al. (2020)
MPS II			Phase II: NCT01962415 (terminated) Phase II: NCT01043640	Vander Lugt et al. (2020)
MPS III			Phase II: NCT00383448	Miller et al. (2011)
MPS VI			Phase II: NCT00668564 Phase II: NCT01043640	Miller et al. (2011)
MPS VII			Phase II: NCT00668564	Miller et al. (2011)
Anakinra				
MPS III			Phase II and III NCT04018755	
Pentosane Polysulfate (PPS)				
MPS I		PPS in MPS I dogs and mice	Phase II: (EudraCt 2014-000,350-11)	Schuchman et al. (2013), Hennermann et al. (2016), Simonaro et al. (2016), Guo et al. (2019)
MSP II			Phase II	Orii et al. (2019)
MPS III		PPS in MPS IIIA mice		Guo et al. (2019)
MPS VI		PPS in MPS VI mice		Frohbergh et al. (2014)
Antioxidant therapy				
Resveratrol				
MPS VII		Resveratrol in MPS VII *Drosophila m.* model		Bar et al. (2018)
Coenzyme Q10				
MPS III	CoQ10 in MPS IIIA and B cells			Matalonga et al. (2014)

## 3 Conclusion

Lysosomal storage appears to have a prominent impact on the inflammatory cytopathology of MPS. Primary and secondary accumulation of undegraded substrates and probably many more factors may initiate a self-propagating innate immune response in MPS—finally leading to inflammation and clinical deterioration. We face a complex interplay between multiple processes on a cellular and humoral level.

Patients affected by MPSs show a wide spectrum of clinical features and disease severity. Understanding the pathomechanism in each MPS type and subtype can reveal insights on how to better treat and manage the disease.

The complexity of the pathogenic cascade in MPS disease provides a number of potential clinical intervention points. Perhaps several different targets have to be addressed together to receive a benefit in clinical features, especially concerning CNS pathology. Combining therapies which target different aspects of the pathogenic cascade has shown neuroprotective effects in a neurodegenerative mouse model of the neurodegenerative LSD Niemann Pick disease type C1 (Williams et al., 2014). This approach may be valuable in MPSs as well. Currently, several promising therapeutic approaches are under investigation, including biologicals and small molecules. Immunomodulatory drugs, within the meaning of repurposing, are emerging tools for specific and/or adjuvant use in patients with MPS disease.

For this purpose, additional and larger clinical trials are needed to investigate benefits of different immunomodulatory agents in MPS. On the other hand, clearly structured and evidence-based individual treatment trials are required, as each MPS patient differs phenotypically among another.

Therefore, the understanding of the role of inflammation in the cytopathology of patients with MPS is necessary to gain new (adjuvant) treatment approaches, causing reduction of symptoms and enhancing quality of life.

## Glossary

AMPK: AMP-activated kinase
APC: antigen presenting cell
BBB: blood brain barrier
BMP: bone morphogenetic proteins
CNS: central nervous system
CoQ10: Coenzyme Q10
CSF: cerebrospinal fluid
C4S: chondroitin 4 sulfate
C6S: chondroitin 6 sulfate
CSPα: cysteine string protein α
DS: dermatan sulfate
ECM: extracellular matrix
EGF: epidermal growth factor
ER: endoplasmatic reticulum
ERT: enzyme replacement therapy
FGF: fibroblast growth factor
GAG: glycosaminoglycan
HA: hyaluronan (hyaluronic acid)
HCQ: hydroxychloroquine
HS: heparan sulfate
HSCT: hematopoietic stem cell transplantation
IL: interleukin
I.V.: intravenous
JIA: juvenile idiopathic arthritis
KS: keratan sulfate
LSD: lysosomal storage disease
MCP: monocyte chemotactic proteins
MPS: mucopolysaccharidoses
MIP: macrophage inflammatory protein
MIF: macrophage migration inhibitory factor
MMP: metalloproteinases
mRNA: messenger ribonucleic acid
mTORC1: mechanistic target of rapamycin complex 1
MW: molecular weight
MYD88: myeloid differentiation primary response 88
PC: pharmacological chaperones
P.O.: per oral
PP: pentosane polysulfate
RNS: reactive nitrogen species
ROS: reactive oxygen species
rRNA: ribosomal ribonucleic acid
SCRT: stop codon read through therapy
SRT: substrate reduction therapy
S.C.: sub cutaneous
TLR4: toll like receptor 4
TNF: tumor necrosis factor
tRNA: transfer ribonucleic acid
UPR: unfolded protein response
